# Evaluating the Impact of Sex and Gender in Brugada Syndrome

**DOI:** 10.19102/icrm.2019.100205

**Published:** 2019-02-15

**Keywords:** Brugada syndrome, gender, sex, testosterone, transgender

## Drs. Wilde and Postema discuss

The Brugada syndrome (BrS) case described by Drs. Sichrovsky and Mittal^1^ is a unique case that may help us to understand the reason for the male preponderance of BrS. Before going into the details, it is important to emphasize that this case is another example of the difficulties that apparently still exist in recognizing a BrS electrocardiogram (ECG) pattern.^2^ An ECG like this one should not be missed anymore!

The patient described is a genetically female patient who had been using testosterone for about 20 years “to live the life of a transgender male.”^1^ He presented with an out-of-hospital cardiac arrest and a BrS type 1 pattern ECG (note, the ECG in Sichrovsky et al.’s **Figure 2** is from five months before the arrest). Unfortunately, we do not have an ECG from before the start of the intramuscular testosterone injections. Importantly, the claim that testosterone converted this female into a symptomatic BrS male, the essence of this case report and the basis for the nice subtitle “Gender trumps sex as a risk factor,” can of course only with confidence be made with the demonstration of a normal ECG prior to the testosterone therapy. Yet, the odds are in favor of the interpretation presented by the authors. Indeed, testosterone serum levels have been shown to impact the degree of right precordial ST-segment amplitude.^3,4^ Interestingly, BrS patients who have undergone orchidectomy lose their type 1 pattern after the procedure,^3^ and androgen deprivation therapy does reduce the ST-segment level in non-BrS patients.^4^ Furthermore, in BrS patients, testosterone levels have been found to be higher as compared with in age-matched controls.^5^

The authors clearly adhere to the “repolarization theory” as the pathophysiological mechanism of the right precordial ST-segment elevation. At this point, we can say that all interventions that increase the early potassium currents, and testosterone may be one of them, also impact in a negative way the safety of conduction.^6^ Hence, the effects of testosterone may also be explained by further deterioration of conduction in the right ventricular outflow tract (RVOT) area.

Finally, we assume that the ectopy shown in this patient is not related to the BrS substrate. Although the origin is in the RVOT area, the coupling interval of ventricular fibrillation (VF)–triggering episodes in BrS patients is shorter as compared with the ectopy in this patient. Earlier studies report a coupling interval of less than 400 ms^7^ and, here, it is 440 ms to 560 ms (**Figure 2** by Sichrovsky et al.). Also, the fact that quinidine was not effective in suppressing the ectopy (while it is very effective in suppressing more serious arrhythmias, as has been described previously in BrS^8^) is in favor of there being a different mechanism for the patient’s ectopy. This potentially explains why the ablation procedure from the endocardial side was successful, whereas the substrate for BrS-related arrhythmias is expected in the epicardial layer.^9^ It is also possible that ablation from the endocardial side does affect the epicardial layer of the RVOT, which, after all, is relatively thin.

In summary, the use of testosterone in this patient most likely contributed to the BrS phenotype and underscores the fact that gender indeed impact the phenotype. The RVOT ectopy is presumably unrelated but may serve as a trigger in the setting of a vulnerable substrate in the epicardial layer of the RVOT region.

Arthur A. M. Wilde, md, phd (a.a.wilde@amc.uva.nl)^1,2^ and Pieter G. Postema, md, phd^1^

^1^Department of Clinical and Experimental Cardiology, Amsterdam University Medical Center, Academic Medical Centre, University of Amsterdam, Amsterdam, the Netherlands

^2^Department of Medicine, Columbia University Irving Medical Center, New York, NY, USA

The authors report no conflicts of interest for the published content.

References1.SichrovskyTCMittalSBrugada syndrome unmasked by use of testosterone in a transgender male: gender trumps sex as a risk factorJ Innov Cardiac Rhythm Manage20191023526352910.19102/icrm.2019.100202PMC7252644324777172.GottschalkBHAnselmDDBrugadaJExpert cardiologists cannot distinguish between Brugada phenocopy and Brugada syndrome electrocardiogram patternsEuropace201618710951100[CrossRef][PubMed]2649815910.1093/europace/euv2783.MatsuoKAkahoshiMSetoSYanoKDisappearance of the Brugada-type electrocardiogram after surgical castration: a role for testosterone and an explanation for the male preponderancePacing Clin Electrophysiol2003267 Pt 115511553[PubMed]1291463810.1046/j.1460-9592.2003.t01-1-00227.x4.EzakiKNakagawaMTaniguchiYGender differences in the ST segment: effect of androgen-deprivation therapy and possible role of testosteroneCirculation J2010741124482454[PubMed]10.1253/circj.cj-10-0221208341865.ShimizuWMatsuoKKokuboYSex hormone and gender difference–-role of testosterone on male predominance in Brugada syndromeJ Cardiovasc Electrophysiol200718415421[CrossRef][PubMed]1739445610.1111/j.1540-8167.2006.00743.x6.WildeAAMPostemaPGDiegoJMThe pathophysiological mechanism underlying Brugada syndrome: depolarization versus repolarizationJ Mol Cell Cardiol2010494543553[CrossRef][PubMed]2065947510.1016/j.yjmcc.2010.07.012PMC29328067.KakishitaMKuritaTMatsuoKMode of onset of ventricular fibrillation in patients with Brugada syndrome detected by implantable cardioverter defibrillator therapyJ Am Coll Cardiol200036516461653[CrossRef][PubMed]1107967110.1016/s0735-1097(00)00932-38.ViskinSWildeAATanHLAntzelevitchCShimizuWBelhassenBEmpiric quinidine therapy for asymptomatic Brugada syndrome: time for a prospective registryHeart Rhythm200963401404[CrossRef][PubMed]1925121910.1016/j.hrthm.2008.11.030PMC30055599.NademaneeKVeerakulGChandanamatthaPPrevention of ventricular fibrillation episodes in Brugada syndrome by catheter ablation over the anterior right ventricular outflow tract epicardiumCirculation20111231212701279[CrossRef][PubMed]2140309810.1161/CIRCULATIONAHA.110.972612

## Dr. Brugada remarks

**Figure 1:**
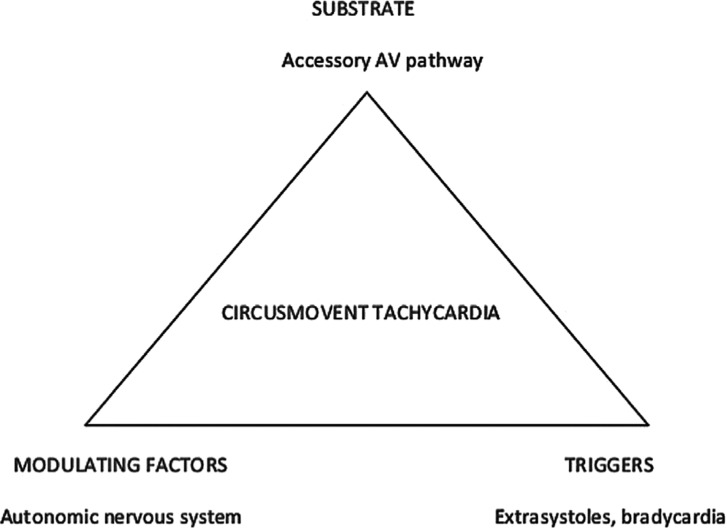
A succinct application of Coumel’s triangle to understand why circusmovement tachycardia in a patient with WPW occurs. See text.

To honor the magnificent contributions of the late Philippe Coumel in the understanding of the mechanisms of cardiac arrhythmias, I coined the term “Coumel’s triangle.”^1^ Coumel’s philosophy on cardiac arrhythmias was based on the concept that a complex situation (a cardiac arrhythmia) is likely the result of multiple causation: the interaction between the substrate of the arrhythmia with the modulating factors facilitates the start of the arrhythmia by way of the appropriate triggers. A presented example can be used to clarify these mechanisms, as follows: a patient with Wolff–Parkinson–White (WPW) syndrome is born with an accessory atrioventricular (AV) pathway (the substrate). The arrhythmia, circusmovement tachycardia, can be triggered by extrasystoles or junctional rhythm during bradycardia. However, the arrhythmia will only initiate and perpetuate if the conduction properties of the different pathways involved in reentry are appropriate. The appropriateness of the conduction properties is modulated, among other ways, by the autonomic nervous system. An application of the Coumel’s triangle in the case of WPW syndrome is shown in **Figure 1**.

**Figure 2:**
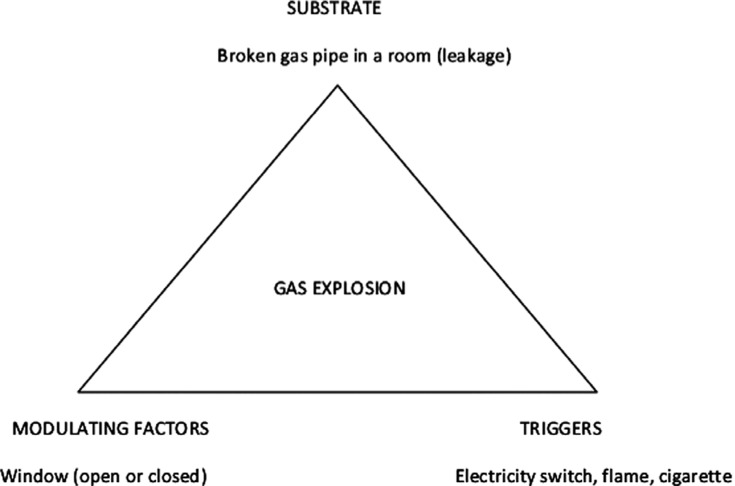
Understanding the factors that play a role in the occurrence of a gas explosion in a room. The substrate is the leakage in the gas pipe. The explosion can only occur if the window is closed, allowing for the accumulation of the gas inside the room. The explosion will occur when triggered by turning on an electricity switch, a flame, or a lighted cigarette in the room.

Coumel’s triangle can be used to understand cardiac arrhythmias and, in fact, it can be applied to understand any phenomenon or event in any domain of knowledge. **Figure 2** shows another succinct application of Coumel’s triangle to understand a gas explosion in a room.

**Figure 3:**
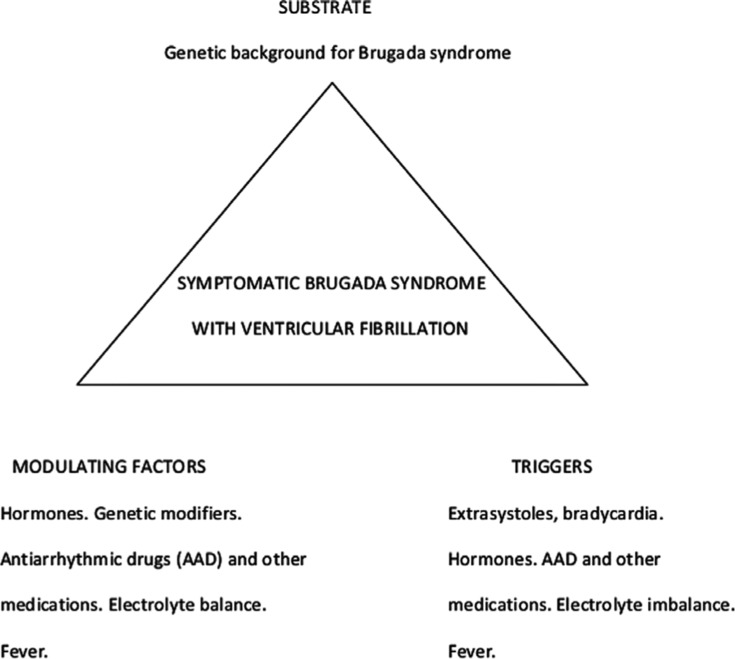
Analysis of the case by Drs. Sichrovsky and Mittal^2^ based on Coumel’s triangle; in this, gender and sex do not compete in importance but rather are both equally relevant.

In the present issue, Drs. Sichrovsky and Mittal present^2^ a phenomenal case of a female-born patient with BrS who has been living as a transgender male via the chronic use of testosterone. According to their description, the patient developed a BrS ECG pattern under this medication. Subsequently, his BrS pattern turned into a full-blown symptomatic syndrome with repeated cardiac arrest. Appropriate protection with an implantable cardioverter-defibrillator was provided to the patient, together with variable quinidine therapy. Symptomatic frequent ventricular extrasystoles prompted endocardial ablation at the RVOT, with a subsequent event-free follow-up. Based on these observations, the authors concluded that “gender trumps sex when it comes to arrhythmia risk in … Brugada syndrome.” Looking at this case with the glasses of Coumel’s triangle, however, I think it is important to state that I disagree with this statement. Biological sex (sex—the substrate) and social sex (gender—a modulating factor) do not compete against each other in importance to trigger VF in BrS. Rather, both are part of the same triangle and are equally relevant **(Figure 3)**. In terms of the triggers, I do believe that the extrasystoles (and the bradycardia) were examples in this patient. Elimination of the ventricular extrasystoles with ablation probably eliminated one of the triggers; however, it is also possible that the authors inadvertently also eliminated the BrS substrate. Successful endocardial (instead of epicardial) ablation of BrS substrate has been reported.^3^

This observation is not surprising, given the very small thickness of the RVOT. Endocardial or epicardial ablation in that area most likely always results in a transmural lesion.^3^ Drs. Sichrovsky and Mittal should be congratulated by their extraordinary observation. This case makes it also clear that testosterone has to be added to the list of drugs to be avoided in confirmed or suspected BrS (www.brugadadrugs.org).

Pedro Brugada, md, phd (pedro@brugada.org)^1^

^1^Cardiovascular Division, University Hospital Brussels (UZ Brussel), Brussels, Belgium

Dr. Brugada reports that he is a consultant for Biotronik and the director of the Biotronik Midwest Bio Alliance for Scientific Growth program.

References1.BrugadaPWellensHAbstracts: 20 years of programmed electrical stimulation of the heart. June 1-5, 1987, Maastricht, The NetherlandsPacing Clin Electrophysiol1987103 Pt 1591622[PubMed]24400112.SichrovskyTCMittalSBrugada syndrome unmasked by use of testosterone in a transgender male: gender trumps sex as a risk factorJ Innov Cardiac Rhythm Manage20191023526352910.19102/icrm.2019.100202PMC7252644324777173.TauberPEMansillaVBrugadaPEndocardial approach for substrate ablation in Brugada syndrome: epicardial, endocardial or transmural substrate?J Clin Exp Res Cardiol201841101113

## Drs. Belhassen and Milman comment

With respect to the present case, we must acknowledge that we have not previously encountered a patient similar to that reported by Drs. Sichrovsky and Mittal.^1^ A thorough Medline search also confirmed the exceptional nature of this case report.

Based on our latest experience with the Survey on Arrhythmic Events (AEs) in BrS (SABRUS),^2,3^ we would like to highlight a few points. The SABRUS gathered 678 patients with BrS and AEs from 23 large centers with prior experience in treating BrS. Fifty-nine (8.7%) of the SABRUS patients were female. Although the patient described in the present case is male in all his being, his genetics are of female origin and so we would like to highlight a few critical points in this regard.

### Arrhythmic events in “females” with Brugada syndrome

As emphasized by the authors, BrS occurs eight to 10 times more frequently in males despite the disease being equally inherited by both genders. The male/female ratio observed in SABRUS was 10.4^3^; however, this ratio varied according to patients’ age and ethnic origin. Considering the 63-year-old Caucasian, genetically female patient at hand yields a male/female ratio of 2.^3^ It is noteworthy that this ratio is much higher in Asian patients of the same age group (ratio of 14).^3^

### Age at onset of initial arrhythmic event

Adult female patients (identified as those aged older than 16 years) included in SABRUS suffered their first AE at a mean age of 49.5 years ± 14.4 years, which was significantly older than that in males (43.0 ± 12.7 years; p = 0.001).^2^ We hypothesized that AE onset in females might correlate with circulating estrogen levels because of a predominance for AEs to occur at ages at which estrogen levels are at their lowest.^2,3^ In the present case, the occurrence of the AE after the age of 60 years could relate more to the marked decrease in estradiol activity rather than to the 20-year use of testosterone.

### Mode of arrhythmic event documentation

When considering the patient’s female genetics, it is not surprising that his primary presentation was of an aborted cardiac arrest, as a majority of females in SABRUS (36/59; 61%) developed their initial AE as a first manifestation of the disease.^3^

### Electrocardiogram at the time of arrhythmic event

Similar to our SABRUS female patients (35/59; 59%),^3^ the ECG at the time of AE did not show a typical type 1 BrS ECG pattern. Interestingly, the latter manifested five months before the event, when the patient was evaluated for an episode of atrial fibrillation.

### Quinidine treatment

The authors used a daily low dose of quinidine (quinidine gluconate ER 324 mg), which was similar to the doses of quinidine (≤ 600 mg daily) reported by Marquez et al.^4^ as effective in preventing AEs in 11 of 14 patients with BrS. It is interesting that such a low dose was apparently effective in both suppressing arrhythmic storms and preventing further AEs during follow-up. This dose is lower than those found to be effective by Anguera et al.^5^ in 19 (66%) of their 29 patients with multiple AEs [quinidine bisulfate (mean dose: 591 ± 239 mg/day) and hydroquinidine (mean dose: 697 ± 318 mg/day)]. In addition, the small dose used in the present paper by Sichrovsky and Mittal^1^ was much lower than the doses necessary to achieve drug efficacy based on serial electrophysiologic testing^6^ (ie, mean dose of 1,406 ± 242 mg of quinidine bisulfate and mean dose of 900 mg of hydroquinidine).

Bearing in mind the present outstanding case report, it seems advisable to closely follow with serial ECGs those subjects using chronic exogenous testosterone supplementation. Although anabolic steroids are known to cause sudden death through ischemic coronary disease,^7,8^ the present case report by Sichrovsky and Mittal highlights the need to consider other possible etiologies such as BrS.

Bernard Belhassen, md (bblhass@gmail.com)^1,2^ and Anat Milman, md, phd^2,3^

^1^Department of Cardiology, Tel-Aviv Sourasky Medical Center, Tel-Aviv, Israel

^2^Sackler School of Medicine, Tel-Aviv University, Tel-Aviv, Israel

^3^The Heart Center, Chaim Sheba Medical Center, Tel Hashomer, Israel

The authors report no conflicts of interest for the published content.

References1.SichrovskyTCMittalSBrugada syndrome unmasked by use of testosterone in a transgender male: gender trumps sex as a risk factorJ Innov Cardiac Rhythm Manage20191023526352910.19102/icrm.2019.100202PMC7252644324777172.MilmanAAndorinASacherFAge of first arrhythmic event in Brugada syndrome: data from the SABRUS (Survey on Arrhythmic Events in Brugada Syndrome) in 678 PatientsCirc Arrhythm Electrophysiol20171012pii: e005222. [CrossRef][PubMed]10.1161/CIRCEP.117.005222292549453.MilmanAGourraudJBAndorinAGender differences in patients with Brugada syndrome and arrhythmic Events: data from a survey on arrhythmic events in 678 patientsHeart Rhythm2018151014571465[CrossRef][PubMed]2990837010.1016/j.hrthm.2018.06.0194.MárquezMFBonnyAHernández-CastilloELong-term efficacy of low doses of quinidine on malignant arrhythmias in Brugada syndrome with an implantable cardioverter-defibrillator: a case series and literature reviewHeart Rhythm201291219952000[CrossRef][PubMed]2305918510.1016/j.hrthm.2012.08.0275.AngueraIGarcía-AlberolaADallaglioPShock reduction with long-term quinidine in patients with Brugada syndrome and malignant ventricular arrhythmia episodesJ Am Coll Cardiol2016671316531654[CrossRef][PubMed]2715069210.1016/j.jacc.2016.01.0426.BelhassenBRahkovichMMichowitzYGlickAViskinSManagement of Brugada syndrome: thirty-three-year experience using electrophysiologically guided therapy with class 1A antiarrhythmic drugsCirc Arrhythm Electrophysiol20158613931402[CrossRef][PubMed]2635497210.1161/CIRCEP.115.0031097.InoueHNishidaNIkedaNThe sudden and unexpected death of a female-to-male transsexual patientJ Forensic Leg Med2007146382386[CrossRef][PubMed]1732046010.1016/j.jcfm.2006.07.0128.LusettiMLicataMSilingardiEReggiani BonettiLPalmiereCPathological changes in anabolic androgenic steroid usersJ Forensic Leg Med201533101104[CrossRef][PubMed]2604850710.1016/j.jflm.2015.04.014

## Dr. Antzelevitch considers

The male predominance in BrS has long been recognized.^1,2^ The recent SABRUS study served to highlight the marked male predominance of the syndrome (91.3%) with minor involvement of pediatric (4.3%) and elderly populations (1.5%). The peak for arrhythmic events occurred at an average age of 42 years.^3^ Women with BrS generally present more benign clinical characteristics, less spontaneous type 1 ECG pattern, and are more likely to be asymptomatic.^1^

The increased risk of males and the protection of females has been the subject of considerable study over the past many years.^2,4–7^ Gender differences in the degree of ST-segment elevation are observed even in individuals with normal hearts. Ezaki et al.^8^ reported on the degree of ST-segment elevation measured in leads V2 and V5, representative of the right and left ventricles, in 640 healthy males and females ranging in age from five years to 89 years. In both sexes, ST-segment elevation was higher in V2 than in V5. At younger ages (ie, 5–12 years), ST-segment elevation was similar and very low in both males and females. It remained at a low level throughout life in females, but increased dramatically in males soon after puberty and subsequently steadily declined with advancing age, suggesting an influence of changing androgen levels. To test this hypothesis, Ezaki et al. evaluated the effects of androgen-deprivation therapy on ST-segment levels in 21 patients with prostate cancer. Notably, androgen-deprivation therapy significantly decreased ST-segment levels in both leads, in support of the hypothesis that testosterone modulates the early phase of ventricular repolarization. Hayashi et al.^9^ reported similar gender- and age-dependent differences in early repolarization in a large cohort of healthy Japanese subjects.

In 2003, Matsuo et al.^10^ reported ECG normalization following orchiectomy in two cases of prostate cancer patients who had displayed a type 1 ST-segment elevation typical of BrS for many years. The disappearance of ST-segment elevation after surgical castration once again suggests an association between the manifestation of the Brugada ECG pattern and testosterone. It is noteworthy that male patients with BrS have been reported to have higher testosterone levels as compared with age-matched controls.^5^ Interestingly, Yamaki et al.^11^ shared that J-wave manifestation in BrS patients peaks in the early morning hours (2:00 am), coincidently in conjunction with peak levels of serum testosterone. These findings may explain why sudden cardiac death in BrS often occurs during sleep in the early morning hours.

Thus, both clinical and basic science studies point to testosterone as the basis for the gender differences in J-wave manifestation underlying the elevation of the ST segment and consequently the expression of the BrS phenotype. However, what is responsible for the testosterone-mediated ST-segment elevation? Our group has previously tested the hypothesis that testosterone influences the expression of *I*_to_. In preliminary studies, Barajas-Martinez et al. reported that chronic exposure to testosterone increases the expression of the transient outward current (*I*_to_) in human-induced, pluripotent-stem-cell-derived cardiomyocytes.^12^ *I*_to_ is responsible for phase I of the action potential and thus for early repolarization of the ventricular myocardium, and its augmentation underlies the accentuation of the J-wave and ST-segment elevation that contributes to the arrhythmogenic substrate in BrS.^13,14^

Inhibition of *I*_to_ has proven to be ameliorative regardless of the cause of BrS. Unfortunately, ion-channel-specific and cardioselective *I*_to_ blockers are not accessible. The best drug currently available in the clinic capable of blocking *I*_to_ is quinidine. Clinical evidence for the effectiveness of quinidine has been reported in numerous studies and case reports.^15–30^ Belhassen et al., who pioneered the use of quinidine in VF,^31^ reported a 90% efficacy in the prevention of VF induction following treatment with quinidine, despite the use of very aggressive protocols of extrastimulation.^31^

In the fascinating case here reported by Sichrovsky and Mittal,^32^ a genetically female patient who presumably was carrying a BrS susceptibility gene variant but who was totally asymptomatic and protected because of her birth gender lost that protection when chronically receiving 200 mg testosterone intramuscularly twice weekly in order to maintain her transgender identity as a male. After experiencing two aborted cardiac arrests at night, the patient was implanted with a dual-chamber implantable cardioverter-defibrillator capable of atrial pacing to avoid the nocturnal bradycardia, which is known to precipitate episodes of ventricular tachycardia/VF. After receiving four appropriate shocks for VF, the patient was placed on quinidine sulfate, which was subsequently changed to quinidine gluconate ER. After a few days, the patient developed angioedema with every dose of quinidine. The addition of the antihistamine ocetrizine subsequently allowed him to tolerate once-daily dosing of quinidine. Although quinidine was effective in suppressing all sustained arrhythmias, a high burden of premature ventricular contractions (PVCs) persisted. The PVCs were successfully eliminated via ablation of the endocardial aspect of the RVOT.

Quinidine’s action to suppress ventricular tachycardia/VF in this case is likely attributable to its inhibition of *I*_to_. The persistence of the PVCs may be due to the relatively low dose of the quinidine prescribed, owing to a desire to minimize angioedema. In addition to its action to block *I*_to_, quinidine also exerts an anticholinergic effect and is an effective inhibitor of the delayed rectifier potassium current (*I*_Kr_), which prolongs the QT interval and is the basis for its use in the treatment of short QT syndrome. Both of these actions may have contributed to its ameliorative action in this patient, particularly because, prior to the administration of quinidine, his QT interval was relatively short (QT interval = 300 ms; corrected QT interval = 335 ms) (see Sichrovsky et al.’s **Figure 2**). Genetic screening was not performed in this case, but the relatively short QT interval suggests the possibility that the case involved a mutation in a calcium channel gene (eg, *CACNA1C, CACNB2*, or *CACNA2D1*).^33^

Charles Antzelevitch, phd (cantzelevitch@gmail.com)^1–3^

^1^Lankenau Institute for Medical Research, Wynnewood, PA, USA

^2^Lankenau Heart Institute, Wynnewood, PA, USA

^3^Sidney Kimmel Medical College of Thomas Jefferson University, Philadelphia, PA, USA

Dr. Antzelevitch acknowledges support from the National Heart, Lung, and Blood Institute (HL138103) and from the Martha and Wistar Morris Fund.

References1.BenitoBSarkozyAMontLGender differences in clinical manifestations of Brugada syndromeJ Am Coll Cardiol2008521915671573[CrossRef][PubMed]1900759410.1016/j.jacc.2008.07.0522.AntzelevitchCAndrogens and male predominance of the Brugada syndrome phenotypePacing Clin Electrophysiol2003267 Pt 114291431[CrossRef][PubMed]1291461710.1046/j.1460-9592.2003.t01-1-00206.x3.MilmanAAndorinAGourraudJBProfile of Brugada syndrome patients presenting with their first documented arrhythmic event: data from the Survey on Arrhythmic Events in Brugada Syndrome (SABRUS)Heart Rhythm2018155716724[CrossRef][PubMed]2932597610.1016/j.hrthm.2018.01.0144.Di DiegoJMCordeiroJMGoodrowRJIonic and cellular basis for the predominance of the Brugada syndrome phenotype in malesCirculation20021061520042011[PubMed]1237022710.1161/01.cir.0000032002.22105.7a5.ShimizuWMatsuoKKokuboYSex hormone and gender difference--role of testosterone on male predominance in Brugada syndromeJ Cardiovasc Electrophysiol2007184415421[CrossRef][PubMed]1739445610.1111/j.1540-8167.2006.00743.x6.FishJMAntzelevitchCCellular and ionic basis for the sex-related difference in the manifestation of the Brugada syndrome and progressive conduction disease phenotypesJ Electrocardiol200336173179[CrossRef][PubMed]10.1016/j.jelectrocard.2003.09.054147166297.VerkerkAOWildersRde GeringelWTanHLCellular basis of sex disparities in human cardiac electrophysiologyActa Physiol (Oxf)20061874459477[CrossRef][PubMed]1686677710.1111/j.1748-1716.2006.01586.x8.EzakiKNakagawaMTaniguchiYGender differences in the ST segment: effect of androgen-deprivation therapy and possible role of testosteroneCirc J2010741124482454[CrossRef][PubMed]2083418610.1253/circj.cj-10-02219.HayashiHMiyamotoAIshidaKPrevalence and QT interval of early repolarization in a hospital-based populationJ Arrhythmia201026212713310.1016/S1880-4276(10)80017-110.MatsuoKAkahoshiMSetoSYanoKDisappearance of the Brugada-type electrocardiogram after surgical castration: a role for testosterone and an explanation for the male preponderancePacing Clin Electrophysiol2003267 Pt 111511153[CrossRef][PubMed]10.1046/j.1460-9592.2003.t01-1-00227.x1291463811.YamakiMSatoNOkadaMA case of Brugada syndrome in which diurnal ECG changes were associated with circadian rhythms of sex hormonesInt Heart J2009505669676[CrossRef][PubMed]1980921510.1536/ihj.50.66912.Barajas-MartinezHHuDUrrutiaJChronic exposure to testosterone increases expression of transient outward current in human induced pluripotent stem cell (hiPSC)-derived cardiomyocytes (CM)Heart Rhythm20131011174110.1016/j.hrthm.2013.09.01213.AntzelevitchC YGJ wave syndrome: Brugada and early repolarization syndromesHeart Rhythm201512818521866[CrossRef][PubMed]2586975410.1016/j.hrthm.2015.04.014PMC473770914.AntzelevitchCYanGXAckermanMJJ-Wave syndromes expert consensus conference report: Emerging concepts and gaps in knowledgeHeart Rhythm20161310e295e324[CrossRef][PubMed]2742341210.1016/j.hrthm.2016.05.024PMC503520815.BelhassenBGlickAViskinSEfficacy of quinidine in high-risk patients with Brugada syndromeCirculation20041101317311737[CrossRef][PubMed]1538164010.1161/01.CIR.0000143159.30585.9016.BelhassenBShapiraIShoshaniDParedesAMillerHLaniadoSIdiopathic ventricular fibrillation: inducibility and beneficial effects of class I antiarrhythmic agentsCirculation198775809816[PubMed]382934310.1161/01.cir.75.4.80917.BelhassenBViskinSAntzelevitchCThe Brugada syndrome: is an implantable cardioverter defibrillator the only therapeutic option?Pacing Clin Electrophysiol2002251116341640[CrossRef][PubMed]1249462410.1046/j.1460-9592.2002.01634.x18.AlingsMDekkerLSadeeAWildeAQuinidine induced electrocardiographic normalization in two patients with Brugada syndromePacing ClinElectrophysiol2001249 Pt 114201422[CrossRef][PubMed]10.1046/j.1460-9592.2001.01420.x1158446819.BelhassenBViskinSAntzelevitchCBrugadaPBrugadaJBrugadaRPharmacologic approach to therapy of Brugada syndrome: quinidine as an alternative to ICD therapy?The Brugada Syndrome: From Bench to Bedside2004Oxford, UKBlackwell Futura20221120.ViskinSWildeAATanHLAntzelevitchCShimizuWBelhassenBEmpiric quinidine therapy for asymptomatic Brugada syndrome: time for a prospective registryHeart Rhythm200963401404[CrossRef][PubMed]1925121910.1016/j.hrthm.2008.11.030PMC300555921.BelhassenBGlickAViskinSExcellent long-term reproducibility of the electrophysiologic efficacy of quinidine in patients with idiopathic ventricular fibrillation and Brugada syndromePacing Clin Electrophysiol2009323294301[CrossRef][PubMed]1927205710.1111/j.1540-8159.2008.02235.x22.MarquezMFBonnyAHernandez-CastilloELong-term efficacy of low doses of quinidine on malignant arrhythmias in Brugada syndrome with an implantable cardioverter-defibrillator: a case series and literature reviewHeart Rhythm20139121995200010.1016/j.hrthm.2012.08.0272305918523.PellegrinoPLDiBMBrunettiNDQuinidine for the management of electrical storm in an old patient with Brugada syndrome and syncopeActa Cardiol2013682201203[CrossRef][PubMed]2370556510.1080/ac.68.2.296728024.ProbstVEvainSGournayVMonomorphic ventricular tachycardia due to brugada syndrome successfully treated by hydroquinidine therapy in a 3-year-old childJ Cardiovasc Electrophysiol200617197100[CrossRef][PubMed]1642641010.1111/j.1540-8167.2005.00329.x25.SchweizerPABeckerRKatusHAThomasDSuccessful acute and long-term management of electrical storm in Brugada syndrome using orciprenaline and quinine/quinidineClin Res Cardiol2010997467470[CrossRef][PubMed]2022183210.1007/s00392-010-0145-726.HasegawaKAshiharaTKimuraHLong-term pharmacological therapy of Brugada syndrome: is J-wave attenuation a marker of drug efficacy?InternMed2014531415231526[CrossRef][PubMed]10.2169/internalmedicine.53.18292503056527.MarquezMFRiveraJHermosilloAGArrhythmic storm responsive to quinidine in a patient with Brugada syndrome and vasovagal syncopePacing Clin Electrophysiol2005288870873[CrossRef][PubMed]1610501810.1111/j.1540-8159.2005.00183.x28.ViskinSAntzelevitchCMarquezMFBelhassenBQuinidine: a valuable medication joins the list of ’endangered species’Europace200791211051106[CrossRef][PubMed]1776179310.1093/europace/eum18129.OhgoTOkamuraHNodaTAcute and chronic management in patients with Brugada syndrome associated with electrical storm of ventricular fibrillationHeart Rhythm200746695700[CrossRef][PubMed]1755618610.1016/j.hrthm.2007.02.01430.RossoRGlickAGliksonMOutcome after implantation of cardioverter defribrillator in patients with Brugada syndrome: a multicenter Israeli study (ISRABRU)Isr Med Assoc J2008106435439[PubMed]1866914231.BelhassenBRahkovichMMichowitzYGlickAViskinSManagement of Brugada syndrome: a 33-year experience using electrophysiologically-guided therapy with class 1A antiarrhythmic drugsCirc Arrhythm Electrophysiol2015861393140210.1161/CIRCEP.115.0031092635497232.SichrovskyTCMittalSBrugada syndrome unmasked by use of testosterone in a transgender male: gender trumps sex as a risk factorJ Innov Cardiac Rhythm Manage20191023526352910.19102/icrm.2019.100202PMC72526443247771733.BurashnikovEPfeifferRBarajas-MartinezHMutations in the cardiac L-type calcium channel associated J wave syndrome and sudden cardiac deathHeart Rhythm201071218721882[CrossRef][PubMed]2081701710.1016/j.hrthm.2010.08.026PMC2999985
